# Effect of Germination on Fatty Acid Composition in Cereal Grains

**DOI:** 10.3390/foods12173306

**Published:** 2023-09-02

**Authors:** Fadwa Al-Taher, Boris Nemzer

**Affiliations:** 1 VDF FutureCeuticals, Inc., Momence, IL 60954, USA; bnemzer@futureceuticals.com; 2 Department of Food Science and Human Nutrition, University of Illinois at Urbana-Champaign, Urbana, IL 61801, USA

**Keywords:** fatty acids, germination, grains, lipids, omega-6, omega-3, sprouts

## Abstract

Sprouted grains are gaining popularity as functional food ingredients. This study aimed to evaluate the lipid and fatty acid composition of eight sprouted grains (millet, amaranth, quinoa, wheat, rye, barley, buckwheat, and oat). The method used was germination for up to 72 h at temperatures ranging from 19–23 °C. In general, the lipid content increased in the various grains sprouted, providing a rich source of polyunsaturated fatty acids. The % oil yield ranged from 1.17 ± 0.02% in sprouted rye to 5.71 ± 0.26% in sprouted amaranth. Germinated oat showed the greatest increase in fat content, 54.3%, compared to the control. Polyunsaturated fatty acids were more prevalent in whole grains (46.9–75.6%) than saturated fatty acids (10.1–25.9%) and increased with sprouting. The primary fatty acids detected in the grains, in order of abundance, were linoleic, oleic, palmitic, linolenic, and stearic acids. Millet sprouts contained the lowest total saturated fatty acids and the highest polyunsaturated fatty acids. Amaranth had the highest amount of saturated fatty acids, while buckwheat contained the lowest quantity of polyunsaturated fatty acids. The lowest omega-6/omega-3 ratio was 7 to 1 in sprouted rye and 8 to 1 in sprouted barley.

## 1. Introduction

The 2020–2025 USDA dietary guidelines recommend Americans consume six one-ounce servings of grains daily, half of which should come from whole grains (USDA dietary guidelines) [1]. Wheat and maize are the most consumed cereal seeds in the American diet. Other consumed grains include amaranth, barley, sorghum, rice, millet, rye, and oats [2,3,4], which are present in bread, pasta, baked goods, breakfast cereals, and other types of foods. Quinoa, buckwheat, and amaranth, gluten-free pseudocereals, have great nutritional value and are important to individuals who have gluten sensitivity and celiac disease [5]. Whole grains contain a high protein content with balanced essential amino acids (methionine and lysine) and a rich source of polyunsaturated fatty acids [6,7,8,9]. The intake of whole grains is linked with an improved body mass index and cholesterol, lower cardiovascular disease and cancer, and reduced diabetes [10].

Sprouted grains are consumed worldwide and are in great demand because, when used as an ingredient, they improve the nutritional content of food [11]. Sprouting increases the dietary value of grains and digestibility and reduces antinutritive compounds such as phytates, tannins, and oxalates, which may inhibit the absorption of minerals such as iron and zinc [12]. Germination results in considerable phytochemical changes in various kinds of grains depending on the grain type and germination conditions, suggesting a fluctuation of nutrients, including remobilization, degradation, and accumulation. A major nutrient modification during the growth of grains is the catabolism of lipids. This process releases energy and produces nitrogen and carbon necessary for the development of the plant [13]. Triglycerides are stored in cereal grains, primarily in the embryo, aleurone, and scutellum. As the lipase enzyme is activated during germination, triacylglycerols are hydrolyzed into free fatty acids and glycerol [10,13].

Studies have demonstrated that the germination process causes different effects on the total fat content of the grains and the fatty acid profile [14,15,16,17,18]. Aparicio-Garcia et al. [14] demonstrated that sprouted oat powder exhibited a similar fatty acid profile as the whole grain with a significant increase in the contents of palmitoleic, linoleic, alpha-linolenic, and cis-11-eicosanoid acids but showed a 3% decrease in the oleic acid content. Peiretti et al. [15] determined that the quinoa plant was mostly enriched with alpha-linolenic acid (385–473 g/kg of total fatty acids), while linoleic acid (146–176 g/kg of total fatty acids) decreased as the plant grew until the shoot stage and then increased. Ortiz et al. [16] noted an increase in stearic acid (49%) and alpha-linolenic acid (24%) and a decrease in oleic acid (6%) in sprouted barley during six days of sprouting compared to the control. Linoleic acid, the main fatty acid in barley, was not affected by germination. Hung et al. [17] did not observe a change in linoleic and linolenic acids during 48 h of germinating wheat. The fatty acid content improved in buckwheat with germination [18].

Omega-6, linoleic acid, and omega-3, linolenic acid, are essential polyunsaturated fatty acids that are not synthesized in the human body but must be obtained from the diet. Individuals with polyunsaturated fatty acids in their diets have a healthier lipid panel, lower incidence of diabetes, and decreased cardiovascular disease [19]. Omega-3 and omega-6 fatty acids provide energy and biological functions that are required by the body [20,21]. Omega-3 may also possibly lower the risk of cancer, cardiovascular diseases, and a variety of mental diseases, including depression, attention-deficit hyperactivity disorder, and dementia [22]. Omega-3 fatty acids are effective in improving the functions of the immune system, reproductive system, and skin [20,21,22]. It is recommended that the omega-6/omega-3 ratio be 2 to 1 to decrease the risk of coronary heart disease [20].

The aim of this study was to evaluate and compare the effect of sprouting on the lipid and fatty acid composition in eight whole grains (millet, amaranth, rye, wheat, quinoa, buckwheat, barley, and oat) to identify the grain with the highest amount of the health-beneficial unsaturated fatty acids and lowest omega-6/omega-3 fatty acid ratio to reduce the risk of cardiovascular disease and other metabolic conditions.

## 2. Materials and Methods

### 2.1. Plant Material

Four types of grains and sprout powders were obtained from different vendors and tested for Study I. Seven varieties of grain seeds were purchased, and the sprouts were germinated in the laboratory for Study II.

#### 2.1.1. Whole Grain and Sprouted Grain Flour (Study I)

Four types of whole grain (raw ingredients) and sprouted grain flour were supplied for this study. Organic sprouted buckwheat flour and its raw ingredient were obtained from Everspring Farms, Canada. Information supplied on the sprouting conditions stated that for buckwheat, water was added to the sprouting vessel only as much as the buckwheat seeds needed to absorb to germinate. Germination was for 24 h at 30 °C. Organic sprouted amaranth powder, organic sprouted quinoa flour, and organic sprouted millet powder and their raw ingredients were acquired from To Your Health Sprouted Flour Company, Fitzpatrick, AL. The grains for these were soaked for 60 min. before germinating. The germination time was 18–24 h in the temperature range of 20.5–22.2 °C.

#### 2.1.2. Whole Grains and Sprouts (Study II)

Seven varieties of whole grain species were purchased and sprouted in the laboratory. Barley seeds were supplied by CZ Grain LLC, Russel, Iowa. Organic whole millet seeds, organic whole oat grain seeds, and organic buckwheat groats were obtained from Handy Pantry, Springfield, IL. Organic Rye seeds were acquired from Thunder Acres, Conway Springs, KS. Wheatgrass seeds, non-GMO, were found at Easy Peasy Plants, Alvin, IL, USA, and amaranth grain, 100% whole seeds, was attained from Food to Live, Brooklyn, NY. All seeds were soaked overnight with distilled water and placed in a 9″ × 9″ plastic, shallow container lined with moist paper towels. Germination was performed once for each of the grains. All seeds germinated from 19–23 °C for 72 h. The germination rate was the following: rye, 80%; oats, 80%; barley, 98%; millet, 98%; buckwheat seeds, 90%; wheat, 100%; and amaranth, 75%. Whole grains (raw ingredients) were used as control samples and ground with an IKA A11 mill, VWR International, LLC., Radnor, PA, USA. All sprouts were then frozen at −80 °C, freeze-dried, and ground to powder with the grinding mill. Four aliquots were collected for the experiment for each of the control and sprouted powders.

### 2.2. Chemicals and Reagents

A FAME (fatty acid methyl ester) mix (37 compounds in hexane ranging from C4:0 to C24:1), 100 mg, was purchased from Sigma-Aldrich, St. Louis, MO, USA. The internal standard, C13:1, methyl-12-tridentate (12-cis-tridecenoic acid methyl ester), 1 g, >99% purity, and other individual FAMEs not included in the FAME 37 mix were bought from Nu-Chek Prep. Inc., Elysian, MN, USA. These are methyl petroselaidate, C18:1T, >99% purity, 500 mg, methyl petroelinate, C18:1, >99% purity, 500 mg, methyl transvaccenate, C18:1T, >99% purity, 500 mg, methyl vaccinate, C18:1, >99% purity, 500 mg, methyl docosatrienoate, C22:3, >99% purity, 100 mg, methyl docosatetraenoate, C22:4, >99% purity, 100 mg, methyl docosapentaaenoate, C22:5 n-3, >99% purity, 500 mg, and methyl docosapentaaenoate, C22:5 n-6, >99% purity, 500 mg. Sodium hydroxide, a 13–15% boron trifluoride methanol complex solution, sodium chloride, 99+ % purity, an ACS reagent, petroleum ether, and diethyl ether were all acquired from Sigma-Aldrich. Methanol, LC/MS grade, and n-hexane, for gas chromatography, were obtained from EMD Millipore, Burlington, MA, USA. Ultra-pure water was obtained from the Milli-Q system, Millipore, Bedford, MA, USA.

### 2.3. Lipid Extraction

The oil content of the raw and germinated flours of the various grains was determined by weighing 2 ± 0.1 g of the grain sample into a Pyrex glass tube, VWR International, LLC, Radnor, PA, extracting with petroleum ether/diethyl ether (1:1 *v*/*v*) and shaking for 1 h on a Wrist Action Shaker, Burrell, Model 75, VWR International, LLC (AOAC Official Method 996.01, 2003.05, modified) [23,24]. The sample was then filtered through a 0.2 µm syringe filter, Phenomenex, Torrance, CA, USA, into another pre-weighed glass tube and evaporated to dryness using nitrogen gas with a vacuum manifold. Extractions were performed four times and then averaged. The % oil yield was calculated as follows: [(m2 − m1/m) × 100]/w, where m2 is the mass of the tube with the extracted oil (g), m1 is the mass of the empty tube (g), m is the mass of the initial sample (g), and w is the dry weight (%).

### 2.4. Fatty Acids Profile

The fatty acid composition of the grain and sprout flours were determined by quantitation of fatty acid methyl esters (FAMEs) using a gas chromatography-flame ionization detector (GC-FID) (AOAC Official Method 996.06, modified) [25,26]. Samples were extracted four times. The internal standard C13:1 was added to the oil samples (10–70 mg), followed by the addition of 2 mL of 0.02 N NaOH in methanol, and was heated at 70 °C for 1 h for saponification. Afterward, 2 mL of 13–15% boron trifluoride in methanol was added to the sample. The sample was heated again at 70 °C for 1 h to convert fatty acids to esters. Afterward, 5 mL of 0.01 M sodium chloride in water and 5 mL of hexane were added to the sample, respectively. The sample was vortexed for 1 min, allowing for a phase separation. The organic layer (top) of the extract containing the FAMEs was transferred into a 2 mL autosampler GC vial to run on the GC-FID.

The FAME content of the grain samples was separated on an Agilent HP-88 column (60 m × 0.25 mm, 0.2 µm) and detected with an Agilent 7890 GC/FID. The FID was run at 250 °C using 45 mL/min of hydrogen and 400 mL/min of air. The injector temperature was 250 °C. The gas chromatography had an oven temperature gradient as follows: initially, the temperature was 140 °C held for 1 min, which was then increased to 180 °C at a rate of 10 °C/min for 5 min and then to 220 °C at a programmable rate of 10 °C/min for 15 min. Helium was used as the carrier gas at a flow rate of 1 mL/min with a split ratio of 50:1. Identification of the analytes was performed by comparing the retention times of the FAMEs found in the samples with those of the FAME (C4–C24) reference standards. Individual fatty acids were calculated from FAMEs using relative response factors and conversion factors to triglyceride equivalents (AOAC Official method 996.06, modified) [25] and as the % of total fatty acids.

### 2.5. Statistical Analysis

Results were reported as the average value ± standard deviation of four independent extractions. Statistical analysis was conducted using Microsoft Excel 365. Analysis of variance (ANOVA) was used to compare the means for the % oil yield and % of total fatty acids for the whole grains and sprout flours. Differences were considered significant at *p* < 0.05.

## 3. Results and Discussion

### 3.1. Lipid Content

The primary lipid groups in cereals are triacylglycerides, glycolipids, and phospholipids. The non-polar triacylglycerides are the most prevalent in cereal grains.

This investigation showed that the lipid content in the eight grains (millet, amaranth, wheat, buckwheat, barley, rye, oat, and quinoa) ranged from 0.80–4.04%. Barley and wheat grains contained the lowest amount of lipids (0.80% and 0.99%, respectively), while the highest quantity was found in amaranth (4.04%) (Table 1).

Chughtai et al. [27] detected 3.7% and 3.8% of fat content in the two different millet grains they tested, while Suma and Urooj [28] determined the amount to be 4.8% and 5.4% in the two millet varieties they examined. Slama et al. [29] reported 5.06% in the pearl millet they extracted. Hlinkova et al. [30] determined the range of oil content to be from 6.4–8.2% in 10 samples of the two species of amaranth (*Amaranthus cruentus* and *Amaranthus hypochondriacus*) extracted. Hlinkova et al. [30] demonstrated differences in oil content that depended on the species. Similarly, He and Corke [31] noticed a variation in oil content in 30 amaranth species ranging from 1.9–8.7%, with an average of 5.0%. Tang et al. [32] determined that quinoa contained 6.58–7.17% oil. Bağci et al. [33] determined that the oil content of rye grains ranged from 0.70–3.92%, similar to our findings. These published oil contents for the various grains align with our findings (Table 1).

The statistical analysis showed significant differences (*p* < 0.05) in the lipid content in the various grains and their respective sprouts. Table 1 shows that the lipid content increased significantly (*p* < 0.05) in three of the four sprouted grain varieties studied when germinated for about 24 h, except in millet, where it decreased (*p* > 0.05). Our study demonstrates that the germination of millet for 18 to 24 h caused a decrease in the fat content of millet from 3.03–2.54%, and when germinated for 72 h, the fat content in millet increased from 2.64–3.89% (Table 1). The lipid content also increased in all grains with significant differences (*p* < 0.05) compared to the controls after germinating for 72 h in the laboratory.

Similar to this study, the lipid content in sprouts increased from 4.41% in the oat raw ingredient to 5.55% in the sprouts grown at 25 °C [34]; 1.62% in the buckwheat control to 2.42% when incubated for three days at 30 °C [35]; and 2.45% in the barley grain to 3.68% when germinated for 6 days at 20 °C [16]. This upsurge may be due to the generation of lipids linked to the seed growth and changes in the configuration occurring after the breakdown of other chemical constituents [36].

On the other hand, Farooqui et al. [37] observed a decrease in the fat content from 2.75% in the barley grain to 2.10% in barley when incubated at 25 °C for 72 h. Rico et al. [38] also observed less fat in barley sprouts than in the control. Kavitha and Parimalavalli [39] reported the fat content of the ungerminated wheat flour to be significantly higher (*p* < 0.05) (1.92 ± 0.66%) than that of the germinated wheat (1.43 ± 0.26%). Suma and Urooj [28] determined that the lipid content for two varieties of millet were 4.8% and 5.4%, which decreased to 3.1% and 4.6%, respectively, in the sprouts when incubated for 3 days and at 20–23 °C. Germination may cause lipids to decrease in grains which is possibly due to using fats as an energy source [39,40,41] or due to increased lipolytic activity allowing the lipase enzyme to release the esterified fatty acids from the triglycerides [42,43]. Sprouting triggers many changes, and this depends on the type of crops, genotype, and growth conditions [44].

Other researchers observed no changes in the lipid content with the sprouting of certain grains. Jiménez et al. [44] did not detect a significant change (*p* < 0.05) in lipid content when germinating quinoa for 24 h at 22 °C to 24 °C and amaranth at 48 h in the dark. Marton et al. [45] and Dhillon et al. [46] determined no changes in the fat content of wheat sprouts when compared to wheat seeds.

### 3.2. Fatty Acids Analysis

Table 2 and Table 3 show the fatty acid profiles of various grains and their sprouted counterparts.

The primary fatty acids found in the grains in this investigation, in order of abundance, were linoleic acid, omega-6 (C18:2 cis), oleic acid (C18:1 cis), palmitic acid (C16:0), linolenic acid, omega-3 (C18:3n-3), and stearic acid (C18:0). Grains were richer in polyunsaturated compared to saturated fatty acids, represented by linoleic and palmitic acids, respectively.

#### 3.2.1. Millet

Millet is an important food staple in Asia and Africa, grown in drought and extreme heat conditions. This cereal grain can be used as a substitute for wheat flour for celiac patients [27].

Millet contained the lowest amount of saturated fatty acid, palmitic acid (C16:0), but had the highest amount of polyunsaturated fatty acid, linoleic acid (C18:2 cis), compared to the other seven fatty acids. Other minor fatty acid components found in millet in this study are presented in Table 2 and Table 3. Our analysis shows that millet had higher concentrations of unsaturated (monounsaturated and polyunsaturated) than saturated fatty acids (Table 2 and Table 3). These results are similar to Chughtai et al. [27] for two varieties of millet (i.e., MB-87 and Sargodha Bajra-2011) and Slama et al. [29] for a pearl millet (*Pennisetum glaucum*) grain. Consequently, the omega-6/omega-3 ratio was 68 and 56 in the two types of millet studied. Slama et al. [29] determined a lower omega-6/omega-3 ratio of 22 in the pearl millet (*Pennisetum glaucum*) grain. This shows that different genotypes of millet grains have different fatty acid compositions.

Germination changed the fatty acid composition in millet. The omega-6 and the omega-3 contents decreased in the sprouted millet compared to the control when incubated from 18–24 h (Table 2) but increased when germinated for 72 h, causing a decrease in the omega-6/omega-3 ratio (Table 3). No other research exists on the fatty acid composition of sprouted millet.

#### 3.2.2. Amaranth

Amaranth, a pseudocereal, is cultivated throughout tropical and moderate regions. Amaranth is used as food for human consumption, as well as animal feed [30]. Amaranth grain contains higher amounts of protein than most other grains, such as maize, rice, sorghum, and rye, as well as soybeans, wheat, peanuts, and corn [30,47].

Our investigation presents the major fatty acids detected in the amaranth grains (Table 2 and Table 3). These agree with those of Hlinkova et al. [30], He et al. [31], and Jahaniaval et al. [48] but reveal a few variations in their amounts. Hlinkova et al. [30] observed 36% and 40.1% of linoleic acid, 28.3% and 23.8% of oleic acid, and 31.3% and 31.5% of palmitic acid in *A. cruentus* and *A. hypochondriacus*, respectively. He et al. [31] noted 36.7% to 55.9% of linoleic acid, 18.7–38.9% of oleic acid, and 19.1–23.4% of palmitic acid in the amaranth grain. Jahaniaval et al. [48] detected 39.4% to 49.1% of linoleic acid, 22.8–31.5% of oleic acid, and 21.4–23.8% of palmitic acid. Hlinkova et al. [30] determined that these differences in the fatty acid content may be due to the genotypes and environmental conditions such as the year of cultivation, soil, and temperature.

Germination caused a decline in the levels of linoleic acid (C18:2), oleic acid (C18:1), and palmitic acid (C16:0) in the amaranth samples sprouted for 72 h at room temperature. Consequently, this lowered the omega-6/omega-3 ratio (Table 2 and Table 3). No other research exists on the fatty composition of sprouted amaranth.

#### 3.2.3. Quinoa

Quinoa (*Chenopodium quinoa* Willd.), a pseudocereal and gluten-free grain, offers a nutritious food ingredient to about 2% of adults and 5% of children with food allergies, such as celiac disease, and allergies to wheat [49]. Quinoa is also beneficial for susceptible groups, such as children, the elderly, athletes, lactose intolerant individuals, and women who may be prone to osteoporosis, diabetes, dyslipidemia, obesity, and anemia [50].

Quinoa grain has the highest amount of linolenic acid, omega-3 (3.63%), compared to the seven other grains that were evaluated. It also has a high content of linoleic acid, omega-6 (61.62%), followed by oleic acid (16.28%) and palmitic acid (10.79%) (Table 2).

When quinoa germinated for 18–24 h at 20.5–22.2 °C, the omega-6 content decreased, oleic acid increased slightly, and palmitic acid decreased somewhat while omega-3 increased (Table 2). Jiménez et al. [44] germinated quinoa under the same conditions and noticed that the linoleic and oleic acid amounts increased but palmitic acid decreased. This decrease in palmitic acid in the quinoa sprout may be due to the lipolytic activity and breakdown of triglycerides and polar lipids into simpler compounds during germination.

In our evaluation, the total saturated and polyunsaturated fatty acids decreased in the germinated quinoa (Table 2). Jiménez et al. [44] also showed a decrease in total saturated fatty acids from 10.58% in the quinoa raw ingredient to 8.59% in the sprouted quinoa and an increase in polyunsaturated fatty acids from 61.53% in the control to 65.52% in the sprouted quinoa.

As the omega-6 decreased and the omega-3 increased in quinoa, the omega-6/omega-3 ratio was reduced from 17 in quinoa seeds to 10 in the sprouts in our study (Table 2). This trend is similar to Jiménez et al. [44], where the omega-6/omega-3 ratio was 5.24 in the quinoa grains and decreased to 4.55 in the germinated quinoa. Tang et al. [32] determined the omega-6/omega-3 ratio to be about 6 to 1. Alvarez-Jubete et al. [5] calculated the omega-6/omega-3 ratio in quinoa to be about 1:6. The omega-6/omega-3 ratio in these investigations is lower than the typical Western diet of 20 to 1 [51].

#### 3.2.4. Buckwheat

Buckwheat (*Fagopyrum esculentum*) is a pseudocereal enriched with health-promoting ingredients such as protein, amino acids, and minerals, which lower cholesterol, possess neuroprotective effects, and have antioxidant activities [35]. Buckwheat can be consumed by individuals who have celiac disease since it is gluten-free [52].

The dominant fatty acids determined in buckwheat were linoleic, oleic, and palmitic acids (Table 2 and Table 3). Buckwheat grain contained the least amount of linoleic acid but was the most abundant in oleic acid compared to the other grains examined.

When buckwheat was incubated for up to 72 h, the linoleic acid content increased (Table 2 and Table 3). This result is consistent with the results of Molska et al. [35] and Kim et al. [53], who also reported an increase when buckwheat was sprouted for 3 days at 30 °C and for 8 days at 25 °C, respectively. Yiming et al. [18], on the other hand, noted a decrease in the amount of linoleic acid when buckwheat germinated for 7 days at 37 °C. Oleic acid remained unchanged while the palmitic acid amount increased when buckwheat was incubated for 18 h to 24 h (Table 2), but both fatty acids decreased when sprouted for 72 h (Table 3). This illustrates that the fatty acid content in buckwheat depends on the environmental conditions applied for germination.

The total saturated and monounsaturated fatty acids remained the same, while the total polyunsaturated fatty acids decreased in buckwheat when sprouted for 18–24 h (Table 2). However, when buckwheat was germinated in the laboratory for 72 h, the total saturated and monounsaturated fatty acids decreased, while the total polyunsaturated fatty acids increased (Table 3). Similar to our study, Molska et al. [35] reported a decrease in saturated fatty acids in buckwheat from 21.2% in the control to 16.8% in the sprout when buckwheat was incubated for 3 days at 30 °C. Polyunsaturated fatty acids increased in buckwheat from 78.5% in the raw ingredient to 83.0% in the germinated buckwheat.

Since there was no change in the omega-6 and omega-3 fatty acid content when buckwheat was germinated for 18–24 h, the omega-6/omega-3 ratio remained the same. On the other hand, when buckwheat was incubated for 72 h, the omega-6/omega-3 ratio was reduced in the sprout (Table 3). Molska et al. [35] and Kim et al. [53] noticed a similar trend. Molska et al. [35] observed a decline in the omega-6/omega-3 ratio, from 19.6 in the buckwheat control to 14.1 in the sprout when germinated for 3 days at 30 °C. Kim et al. [53] noted that the omega-6/omega-3 ratio decreased from 14.1 in the buckwheat control to 2.7 in the buckwheat sprout when incubated for 8 days at 25 °C. This may be due to a longer time for germination.

#### 3.2.5. Wheat

Wheat (*Triticum aestivum* L.) is a cereal grain that is grown and consumed throughout the world [54]. Whole wheat grain is an important source of several nutrients and dietary fiber. It can lower the risk of cancer, diabetes, and heart disease [55].

The most prevalent fatty acids detected in wheat in this study (Table 3), comparable to Hung et al. [17], in order of highest to lowest abundance, are linoleic, palmitic, oleic, and linolenic acids. Wheat contained the most linoleic acid after millet powder (Table 3).

In our investigation, germinating wheat modified the fatty acids. Linoleic and palmitic acids increased when wheat was sprouted, but oleic acid was reduced in wheat (Table 3). Similarly, Márton et al. [45] noticed that after 3 days of geminating wheat at 20 °C, the amounts of palmitic and linoleic acids increased in the sprouted wheat while the levels of oleic acid decreased. Hung et al. [17] reported no change in the fatty acid content of wheat when germinating for 48 h. Ozturk et al. [56] observed an increase in linolenic acid, omega-3, while the amount of oleic and linoleic acids decreased when sprouting two varieties of wheat (*cvs* Demir 2000 and Konya 2002).

The total polyunsaturated fatty acids and the total saturated fatty acids increased with sprouting in this study, while the monounsaturated fatty acids decreased when germinating wheat for 72 h (Table 3). However, Hung et al. [17] did not observe changes in the total polyunsaturated, saturated, and monounsaturated fatty acids in wheat when germinated for 48 h.

Since the linoleic acid, omega-6, and linolenic acid, omega-3 increased, when sprouting wheat for 72 h, the omega-6/omega-3 ratio was reduced (Table 3). However, the omega-6/omega-3 ratio was not changed in wheat when germinated for 48 h [17].

#### 3.2.6. Barley

Barley (*Hordeum vulgare)* is a cereal grown in mild temperatures [57]. It has been used as animal feed and as a raw ingredient for beer, distilled beverages, foods, and malted beverages [58]. Barley is an attractive functional ingredient reducing blood cholesterol and glycemic index [59].

The predominant fatty acids detected in barley, from the most to the least abundant, are linoleic, palmitic, oleic, linolenic, and stearic acids (Table 3). Barley had the highest amount of saturated fatty acid, palmitic acid (C16:0), compared to the other seven grains evaluated.

Ortiz et al. [16] observed an increase in linoleic acid (C18:3n-3) and stearic acid (C18:0) by 49% and 24%, respectively, (*p* < 0.01) in sprouted barley after six days of germination, but a 6% decrease in oleic acid (C18:1) (*p* < 0.05). However, our study did not observe a significant difference (*p* > 0.05) in the linoleic acid (C18:3n-3) content when barley was germinated for three days, but approximately a 9% increase in stearic acid (C18:0) and 20.9% decrease in oleic acid (C18:1) (*p* < 0.05). Our findings agree with Ortiz et al. [16] who did not detect a significant difference (*p* > 0.05) in the linoleic acid content, the predominant fatty acid when barley was sprouted.

The total monounsaturated and polyunsaturated fatty acids decreased when barley was sprouted due to the major components, oleic acid (C18:1) and linoleic acid (C18:2), respectively. The total saturated fatty acids increased due to a slight increase in stearic acid (C18:0) (Table 3). There was no significant difference (*p* > 0.05) in linoleic acid (C18:2) for barley but there was a significant increase (*p* < 0.05) in linolenic acid (C18:3n-3) for sprouted barley. Therefore, the omega-6/omega-3 ratio was reduced from 16 for the control to 10 for the sprouted barley. Ortiz et al. [16] did not observe a significant difference (*p* > 0.05) in the linoleic acid (C18:2) content when sprouting but did notice a significant increase (*p* < 0.05) in the linolenic acid (C18:3n-3) content. This resulted in an improved omega-6/omega-3 ratio of 8.40 in the sprout compared to 12.5 in the raw ingredient.

#### 3.2.7. Rye

Rye (*Secale cereale*) is grass grown as a cereal grain, a crop to cover the soil, and plant matter for livestock. It is related to both wheat and barley [60].

Our study shows that rye is enriched with linoleic acid (C18:2 cis), followed by palmitic acid (C16:0), oleic acid (C18:1 cis), and linolenic acid (C18:3n-3) (Table 3). Bağci et al. [33] found that palmitic, stearic, oleic, and linoleic acids were the most abundant in the rye grains. The palmitic acid content of oil samples ranged from 10.82% to 22.43%, whereas the stearic acid amount ranged from 1.25–7.74%. Additionally, the oleic acid quantity varied between 20.61% and 37.86%. The amount of linoleic acid in rye oils ranged from 18.91 to 54.00%, while the linolenic acid content was between 2.43 and 8.34%. Our results were in close agreement with Bağci et al. [33]. Our evaluation showed palmitic acid to be approximately 14.34% and 1.17% for stearic acid in rye. Conversely, our results were lower for oleic acid (9.50%) and higher for linoleic (62.86%) and linolenic (9.19%) acids (Table 3).

When rye was germinated, palmitic acid content significantly increased (*p* < 0.05), but the stearic and linolenic acid content remained the same. The oleic and linoleic acid quantities decreased significantly (*p* < 0.05) when rye was sprouted (Table 3). No other studies have examined the fatty acid composition in germinated rye.

Our study determined that the total saturated fatty acids and the monounsaturated fatty acids decreased from 15.51–15.93% and 12.33–10.05%, respectively, while the total polyunsaturated fatty acids increased from 72.05–74.04% when rye was germinated (Table 3). Bağci et al. [33] observed the total amount of saturated fatty acids in rye oil samples ranged from 15.57 to 34.38%, which is similar to our results. However, Bağci et al. [33] determined that the total polyunsaturated fatty acid amount was higher than the total monounsaturated fatty acids varying from 21.44–61.43% and 21.37–45.15%, respectively. These differences may be due to the environment, genotype, cultivating conditions, and rye varieties [61]. Our evaluation showed that the total amount of monounsaturated fatty acids was lower, while the total polyunsaturated fatty acids were higher in rye grains. The quantity of omega-6 increased slightly in rye when it was incubated, while the omega-3 content remained the same. Therefore, the omega-6/omega-3 ratio was unchanged in the sprouted rye.

#### 3.2.8. Oat

Oat (*Avena sativa*) are cereal grains consumed by humans in the form of oatmeal, rolled oats, oat bran, oatmeal, oat flour, and oat flakes [62]. They may be added to porridge, cereals, granola bars, and baked goods, such as bread and biscuits [63]. Oats can also be used as livestock feed [62]. They are non-allergenic and a good source of dietary fiber, mainly β-glucan, proteins, minerals, and other nutrients [63]. Oats contain nutrients associated with lower blood cholesterol [62]. They are effective against celiac disease and can treat diabetes and cardiovascular diseases [64].

In our investigation, linoleic acid was the predominant fatty acid in the oat grain powder, followed by similar amounts of oleic and palmitic acids and smaller amounts of stearic and linolenic acids (Table 3). Aparicio-Garcia et al. [14] observed that non-germinated oats had the highest content of linoleic acid, with the same quantity of oleic and stearic acids and the least amount of palmitic acid. Unlike our study, Aparicio-Garcia et al. [14] determined that omega-3 (linolenic acid) was present in a smaller amount in oats. Oat grain and sprouted oat flour had a different fatty acid composition than what we observed in our investigation.

Our research showed that sprouted oat powders displayed a similar fatty acid profile as the control. The contents of the linoleic, oleic, and stearic acid did not significantly change (*p* > 0.05) when the oats were germinated. On the other hand, the amounts of palmitic and linolenic acids increased significantly (*p* < 0.05) in sprouted oat (Table 3). Unlike the results in our study, Aparicio-Garcia et al. [14] detected a significant increase (*p* < 0.05) in linoleic and linolenic acids in the sprouted oat powder when germinated at 18 °C for 4 days in the dark compared to the raw ingredient. However, there was a significant decrease in oleic acid (*p* < 0.05) when the oat was sprouted compared to the oat flour, whereas the amount of palmitic acid remained the same. During germination, desaturase enzymes may be activated, converting oleic acid to linoleic and linolenic acids [65].

The total polyunsaturated fatty acids were more abundant in oat flour than the total monounsaturated and saturated fatty acids and increased with the germination of the oats. The total monounsaturated and saturated fatty acids remained the same with the sprouting of the oats (Table 3). Since the omega-6 content remained unchanged and the omega-3 quantity increased slightly in the sprouted oats, the omega-6/omega-3 ratio decreased (Table 3). On the other hand, Aparicio-Garcia et al. [14] calculated a higher omega-6/omega-3 ratio of 26.2 in the oat control, which decreased to 22.6 in the sprouts when incubated at a low temperature of 18 °C for 4 days. This was due to the quantity of linoleic and linolenic acids increasing slightly in the sprouted oat.

In summary, the concentration of unsaturated fatty acids was higher in grains compared to saturated fatty acids. The total saturated fatty acid concentrations ranged from 10.1–25.9% of the total fatty acids in the eight grains (amaranth, millet, quinoa, wheat, oat, rye, buckwheat, and barley) evaluated. Amaranth had the highest amount, followed by buckwheat and oat. The amounts decreased in these sprouted grains. The lowest amount of the total saturated fatty acids was in millet, which remained the same when germinated for 72 h. The total monounsaturated fatty acids varied from 9.5–32.3% of the total fatty acids in the eight grains studied. The buckwheat grain had the highest amount of the total monounsaturated fatty acids with no change in the content when sprouted compared to all other grains. The barley grain had the least monounsaturated fatty acids, which decreased when germinating. The total polyunsaturated fatty acids varied between 46.9% and 75.6% in the whole grains. From the highest to the lowest abundance of total polyunsaturated fatty acids in the grains were millet (75%), rye (72%), wheat (70%), barley (67%), quinoa (65%), amaranth (59%), oat (53%), and buckwheat (47%). Individual fatty acids either decreased, increased, or remained the same in sprouted grains depending on the initial germination and possible lipase activity in the whole grains (Table 2 and Table 3). Benincasa et al. [66] noted that the fatty acid content depends on pre-germination treatments and lipase activity in the grain tissue and some fatty acids either increased or decreased during the germination time. Fatty acid composition depends on the type of grain, germination conditions such as time and temperature, and environmental conditions.

The omega-6 fatty acid surged in buckwheat but was reduced in amaranth, millet, and quinoa when germinated for 18–24 h (Table 2). At 72 h of germination, the omega-6 fatty acid content increased in rye, buckwheat, wheat, millet, and amaranth but decreased in barley and remained the same in oat compared to the whole grains (Table 3). Millet contained the most omega-6, decreasing when germinated for 18–24 h but increasing at 72 h. The omega-3 fatty acid content decreased in millet and increased in amaranth and quinoa but did not change in buckwheat when sprouted for 18–24 h. However, at 72 h of incubation, the omega-3 fatty acid increased in amaranth, oat, wheat, and millet but remained the same as the controls in barley and rye. Rye was the most abundant in the omega-3 fatty acid content, having similar amounts when germinated. The highest omega-6/omega-3 ratio was in the amaranth and millet flours and the lowest was in rye and barley, which decreased with germination, except for rye (Table 2 and Table 3).

## 4. Conclusions

Sprouted grains are gaining popularity as functional foods as they have the potential to reduce risks associated with chronic diseases and other metabolic conditions, such as cardiovascular disease, cancer, and diabetes. It has been found that sprouting can boost the nutritional value of grains. Findings from this study also demonstrate that the sprouting of grains caused changes in their lipid and fatty acid compositions, resulting in potentially increased health benefits.

The lipid content increased in all eight grains (amaranth, millet, quinoa, wheat, oat, rye, buckwheat, and barley) investigated when germinated for up to 72 h. However, the lipid concentration decreased in millet when incubated for 24 h but increased when grown for 72 h. This may be due to either the variation in the two different types of millet used in this study or the growing conditions. The most lipid among the sprouted grains was found in amaranth and the lowest in rye. The lipid content increased the most in sprouted oat at 54.3% when compared to the control.

Polyunsaturated fatty acids were more prevalent in whole grains than saturated fatty acids and increased with germination. Saturated fatty acids decreased, while monounsaturated and polyunsaturated fatty acids increased, causing a varying effect on the omega-6/omega-3 fatty acid ratio. Unsaturated fatty acids lower the risk of heart attacks.

Millet sprouts contained the lowest total saturated fatty acids and the highest polyunsaturated fatty acids. Amaranth had the highest amount of saturated fatty acids, while buckwheat contained the lowest amount of polyunsaturated fatty acids. The lowest omega-6/omega-3 ratio was in the sprouted rye and barley at 7:1 and 8:1, respectively, which is close to the recommended ratio of 2:1 to reduce the risk of coronary disease, while the highest omega-6/omega-3 ratio was found in the millet sprout at 506:1 and the amaranth sprout at 54:1.

The difference in the fatty acid composition in sprouted grains may result in various health effects on humans when consumed. Future research should focus on determining the fatty acid content in grains for longer germination times and at higher temperatures. Additional studies are also required to compare the fatty acid composition in different genotypes of the same species. Cultivation conditions could also affect the total fatty acids and their composition, while different germination conditions could affect the lipase activity (which should be measured in future studies) and consequently the fatty acid and fatty acid composition.

## Figures and Tables

**Table 1:** Lipid content (%) in whole grains and sprouted grains.

	Study I ^1^		Study II ^2^	
	% Lipid Content in Whole Grains (n = 4)	% Lipid Content in Sprouted Grains (n = 4)	% Lipid Content in Whole Grains (n = 4)	% Lipid Content in Sprouted Grains (n = 4)
Millet	3.03 ± 0.31 ^a^	2.54 ± 0.14 ^b^	2.64 ± 0.07 ^a^	3.89 ± 0.06 ^b^
Amaranth	3.43 ± 0.39 ^a^	5.20 ± 0.76 ^b^	4.04 ± 0.15 ^a^	5.71 ± 0.26 ^b^
Quinoa	3.38 ± 0.31 ^a^	4.62 ± 0.70 ^b^	N/A	N/A
Buckwheat	1.47 ± 0.16 ^a^	2.27 ± 0.14 ^b^	1.21 ± 0.06 ^a^	3.54 ± 0.27 ^b^
Barley	N/A		0.80 ± 0.05 ^a^	1.78 ± 0.150 ^b^
Oat			1.76 ± 0.13 ^a^	3.85 ± 0.56 ^b^
Rye			0.67 ± 0.06 ^a^	1.17 ± 0.02 ^b^
Wheat			0.99 ± 0.08 ^a^	1.31 ± 0.10 ^b^

Results are the mean ± standard deviation of four replicates. Different letters in the same row (^a^,^b^) denote significant differences for whole grain and sprouted grain samples (*p* < 0.05). N/A means not applicable since the grain was not tested. ^1^ Study I—germination at 20.5–22.2 °C for 18–24 h. ^2^ Study II—germination at 19–23 °C for 72 h.

**Table 2:** Fatty acid profile (% total fatty acids) in whole grain and sprouted grain flour (Study I).

	Millet Flour	Millet Sprout Flour	Amaranth Flour	Amaranth Sprout Flour	Quinoa Flour	Quinoa Sprout Flour	Buckwheat Flour	Buckwheat Sprout Flour
C14:0 Myristic acid %	N/A	N/A	0.20 ± 0.005 ^a^	0.20 ± 0.006 ^a^	0.21 ± 0 ^a^	0.13 ± 0.01 ^b^	N/A	N/A
C15:0 Pentadecanoic acid %	N/A	N/A	0.07 ± 0 ^a^	0.07 ± 0.01 ^a^	0.36 ± 0.01	0	N/A	N/A
C16:0 Palmitic acid %	7.60 ± 0.01 ^a^	9.25 ± 0.25 ^b^	20.69 ± 0.11 ^a^	19.10 ± 0.33 ^b^	10.79 ± 0.13 ^a^	10.58 ± 0.07 ^b^	17.91 ± 0.25 ^a^	18.13 ± 0.03 ^b^
C16:1 Palmitoleic acid %	0.15 ± 0 ^a^	0.19 ± 0.01 ^b^	N/A	N/A	N/A	N/A	0.22 ± 0.01 ^a^	0.20 ± 0 ^b^
C18:0 Stearic acid %	2.59 ± 0.06 ^a^	2.21 ± 0.03 ^b^	4.34 ± 0.10 ^a^	4.00 ± 0.12 ^b^	1.06 ± 0.02 ^a^	0.67 ± 0.01 ^b^	2.74 ± 0.03 ^a^	2.76 ± 0.02 ^a^
C18:1 cis Oleic acid %	11.94 ± 0.10 ^a^	13.85 ± 0.16 ^b^	16.99 ± 0.02 ^a^	15.98 ± 0.18 ^b^	16.28 ± 0.09 ^a^	16.39 ± 0.13 ^b^	26.07 ± 0.03 ^a^	26.09 ± 0.04 ^a^
C18:1 cis Vaccenic acid %	0.78 ± 0.01 ^a^	1.02 ± 0.01 ^b^	1.42 ± 0.04 ^a^	1.21 ± 0.03 ^b^	1.36 ± 0.03 ^a^	1.13 ± 0.02 ^b^	1.61 ± 0.02 ^a^	1.50 ± 0.01 ^b^
C18:2 cis Linoleic acid Omega-6%	73.64 ± 0.26 ^a^	70.97 ± 0.17 ^b^	57.64 ± 0.08 ^a^	54.66 ± 0.72 ^b^	61.62 ± 0.12 ^a^	58.94 ± 0.40 ^b^	38.93 ± 0.26 ^a^	39.46 ± 0.21 ^b^
C18:3n-6 Gamma-Linolenic acid %	0.47 ± 0.02 ^a^	0.41 ± 0.01 ^b^	0.60 ± 0.01 ^a^	0.46 ± 0.03 ^b^	0.25 ± 0.01 ^a^	0.29 ± 0.01 ^b^	1.19 ± 0.05 ^a^	1.19 ± 0.02 ^a^
C18:3n-3 Linolenic acid Omega-3%	1.08 ± 0.02 ^a^	0.14 ± 0 ^b^	0.88 ± 0.01 ^a^	1.01 ± 0.01 ^b^	3.63 ± 0.04 ^a^	5.76 ± 0.09 ^b^	2.05 ± 0.02 ^a^	2.06 ± 0.02 ^a^
C20:1 Eicosenoic acid %	0.53 ± 0.02 ^a^	0.60 ± 0.01 ^b^	0.28 ± 0 ^a^	0.29 ± 0.01 ^a^	2.22 ± 0.02 ^a^	2.16 ± 0.05 ^a^	4.44 ± 0.11 ^a^	4.32 ± 0.01 ^a^
C22:0 Docosanoic acid %	0.56 ± 0.03 ^a^	0.54 ± 0.01 ^a^	0.42 ± 0.01 ^a^	0.33 ± 0.03 ^b^	0.74 ± 0.01 ^a^	0.79 ± 0.03 ^b^	2.59 ± 0.15 ^a^	2.58 ± 0.06 ^a^
C24:0 Lignoceric acid %	0.40 ± 0.03 ^a^	0.57 ± 0.01 ^b^	0.24 ± 0.01 ^a^	0.22 ± 0.02 ^a^	0.32 ± 0.01 ^a^	0.36 ± 0.02 ^b^	1.51 ± 0.18 ^a^	1.52 ± 0.01 ^a^
∑ Saturated fatty acids	11.15	12.57	25.89	23.85	13.12	12.53	24.75	24.99
∑ Monounsaturated fatty acids	13.4	15.66	18.69	17.48	19.86	19.68	32.34	32.11
∑ Polyunsaturated fatty acids	75.19	71.52	59.12	56.13	65.5	64.99	42.17	41.52
omega-6: omega-3	68	506	66	54	17	10	19	19

Data are the mean ± standard deviation of four replicates. Different letters in the same row (^a^ and ^b^; whole grains and sprouts) indicate significant differences (*p* < 0.05). N/A means not applicable as there were no fatty acids detected and, therefore, there was no quantitation.

**Table 3:** Fatty acid profile (% total fatty acids) in whole grain and sprouted grain powders (Study II).

	**Millet Powder**	**Millet Sprout Powder**	**Amaranth Powder**	**Amaranth Sprout Powder**	**Buckwheat Powder**	**Buckwheat Sprout Powder**	**Barley Powder**	**Barley** **Sprout Powder**
C14:0 Myristic acid %	N/A	N/A	0.20 ± 0.01 ^a^	0.21 ± 0.006 ^a^	N/A	N/A	N/A	N/A
C15:0 Pentadecanoic acid %	N/A	N/A	0.08 ± 0.01 ^a^	0.09 ± 0.003 ^b^	N/A	N/A	N/A	N/A
C16:0 Palmitic acid %	7.55 ±0.03 ^a^	8.15 ± 0.11 ^b^	20.72 ± 0.20 ^a^	19.37 ± 0.07 ^b^	17.77 ± 0.13 ^a^	15.94 ± 0.27 ^b^	21.61 ± 0.05 ^a^	21.81 ± 0.65 ^a^
C16:1 Palmitoleic acid %	0.16 ± 0	0	0.09 ± 0.01 ^a^	0.1 ± 0.003 ^b^	N/A	N/A	N/A	N/A
C17:0 Heptadecanoic acid%	N/A	N/A	0.13 ± 0.01 ^a^	0.12 ± 0.005 ^a^	N/A	N/A	N/A	N/A
C18:0 Stearic acid %	1.72 ± 0.01 ^a^	1.87 ± 0.04 ^b^	0.08 ± 0	0.08 ± 0	2.32 ± 0.02 ^a^	2.35 ± 0.01 ^b^	1.18 ± 0.01 ^a^	1.29 ± 0.04 ^b^
C18:1 cis Oleic acid %	12.39 ± 0.02 ^a^	10.90 ± 0.07 ^b^	16.93 ± 0.15 ^a^	14.72 ± 0.07 ^b^	22.56 ± 0.02 ^a^	20.70 ± 0.08 ^b^	7.51 ± 0.05 ^a^	6.09 ± 0.08 ^b^
C18:1 cis Vaccenic acid %	0.94 ± 0.03 ^a^	0.89 ± 0.02 ^a^	1.40 ± 0.06 ^a^	1.25 ± 0.03 ^b^	1.48 ± 0.09 ^a^	1.43 ± 0.01 ^b^	0.95 ± 0.01 ^a^	0.81 ± 0.09 ^b^
C18:2 cis Linoleic acid Omega-6%	73.98 ± 0.08 ^a^	74.33 ± 0.17 ^b^	56.70 ± 0.20 ^a^	59.50 ± 0.09 ^b^	43.26 ± 0.19 ^a^	47.65 ± 0.03 ^b^	60.66 ± 0.09 ^a^	58.59 ± 5.64 ^a^
C18:3n-6 Gamma-Linolenic acid %	0.36 ± 0.01 ^a^	0.35 ± 0.01 ^a^	0.63 ± 0.04 ^a^	0.59 ± 0.01 ^a^	0.98 ± 0.02 ^a^	1.01 ± 0.02 ^a^	N/A	N/A
C18:3n-3 Linolenic acid Omega-3%	1.31 ± 0.01 ^a^	2.19 ± 0.02 ^b^	0.89 ± 0.01 ^a^	2.50 ± 0.02 ^b^	2.68 ± 0.03 ^a^	3.77 ± 0.04 ^b^	7.03 ± 0.07 ^a^	7.52 ± 0.96 ^a^
C20:1 Eicosenoic acid %	0.53 ± 0.01 ^a^	0.50 ± 0.02 ^a^	0.29 ± 0.01 ^a^	0.28 ± 0 ^b^	4.61 ± 0.05 ^a^	4.67 ± 0.08 ^a^	1.08 ± 0.01 ^a^	0.84 ± 0.07 ^b^
C20:2 Eicosadienoic acid%	N/A	N/A	0.10 ± 0.01 ^a^	0.11 ± 0.01 ^a^	0.23 ± 0.01	0	N/A	N/A
C22:0 Docosanoic acid %	0.48 ± 0.01 ^a^	0.51 ± 0.02 ^a^	0.44 ± 0.04 ^a^	0.53 ± 0.01 ^b^	2.27 ± 0.09 ^a^	2.50 ± 0.09 ^b^	N/A	N/A
C22:1 Erucic acid%	N/A	N/A	N/A	N/A	0.27 ± 0.01	0	N/A	N/A
C23:0 Tricosanoic acid%	N/A	N/A	0.13 ± 0.01 ^a^	0.12 ± 0.01 ^a^	N/A	N/A	N/A	N/A
C24:0 Lignoceric acid %	0.35 ± 0.01 ^a^	0.44 ± 0.01 ^b^	0.27 ± 0.03 ^a^	0.36 ± 0.01 ^b^	1.39 ± 0.10	0	N/A	N/A
C22:5n-3 Docosapentaenoic acid%	N/A	N/A	0.91 ± 0.10 ^a^	0.09 ± 0.04 ^b^	N/A	N/A	N/A	N/A
∑ Saturated fatty acids	10.1	10.97	22.05	20.88	23.96	20.79	22.79	23.1
∑ Monounsaturated fatty acids	14.02	12.29	18.71	16.35	28.92	26.80	9.54	7.74
∑ Polyunsaturated fatty acids	75.65	76.87	59.13	62.59	46.92	52.43	67.69	66.11
omega-6: omega-3	56	34	64	24	16	13	9	8
Data are the mean ± standard deviation of four replicates. Different letters in the same row (^a^ and ^b^; whole grains and sprouts) indicate significant differences (*p* < 0.05). N/A means not applicable as those fatty acids were not detected so there were no amounts to quantitate								
	**Wheat** **Powder**		**Wheat Sprout Powder**		**Rye Powder**	**Rye Sprout Powder**	**Oat Powder**	**Oat Sprout Powder**
C14:0 Myristic acid %	N/A		N/A		N/A	N/A	0.47 ± 0.02 ^a^	0.43 ± 0.04 ^a^
C15:0 Pentadecanoic acid %								
C16:0 Palmitic acid %	17.51 ± 0.07 ^a^		20.28 ± 2.53 ^a^		14.34 ± 0.08 ^a^	14.82 ± 0.19 ^b^	20.88 ± 0.05 ^a^	19.82 ± 0.29 ^b^
C16:1 Palmitoleic acid %	N/A		N/A		N/A	N/A	0.34 ± 0	0
C17:0 Heptadecanoic acid%	N/A		N/A		N/A	N/A		
C18:0 Stearic acid %	1.13 ± 0.04 ^a^		1.35 ± 0.25 ^b^		1.17 ± 0.04 ^a^	1.11 ± 0.01 ^a^	2.32 ± 0.03 ^a^	2.32 ± 0.01 ^a^
C18:1 cis Oleic acid %	8.45 ± 0.06 ^a^		6.86 ± 0.06 ^b^		9.50 ± 0.10 ^a^	7.39 ± 0.04 ^b^	20.79 ± 0.06 ^a^	20.87 ± 0.03 ^a^
C18:1 cis Vaccenic acid %	1.05 ± 0.05		0		1.33 ± 0.01 ^a^	1.27 ± 0.06 ^a^	1.17 ± 0.02 ^a^	1.20 ± 0.02 ^a^
C18:2 cis Linoleic acid Omega-6%	66.44 ± 0.03 ^a^		67.07 ± 1.34 ^a^		62.86 ± 0.22 ^a^	64.52 ± 0.31 ^b^	50.69 ± 0.36 ^a^	50.86 ± 0.14 ^a^
C18:3n-6 Gamma-Linolenic acid %	N/A		N/A		N/A	N/A	0.15 ± 0	0.15 ± 0
C18:3n-3 Linolenic acid Omega-3%	4.22 ± 0.04 ^a^		6.73 ± 0.23 ^b^		9.19 ± 0.11 ^a^	9.52 ± 0.30 ^a^	2.08 ± 0.04 ^a^	2.75 ± 0.06 ^b^
C20:1 Eicosenoic acid %	1.21 ± 0.03		0		1.50 ± 0.02 ^a^	1.39 ± 0.06 ^b^	1.03 ± 0.01 ^a^	1.14 ± 0.02 ^b^
C20:2 Eicosadienoic acid%	N/A		N/A		N/A	N/A	N/A	N/A
C22:0 Docosanoic acid %	N/A		N/A		N/A	N/A	N/A	N/A
C22:1 Erucic acid%	N/A		N/A		N/A	N/A	N/A	N/A
C23:0 Tricosanoic acid%	N/A		N/A		N/A	N/A	N/A	N/A
C24:0 Lignoceric acid %	N/A		N/A		N/A	N/A	0.31 ± 0.03 ^a^	0.53 ± 0.04 ^b^
C22:5n-3 Docosapentaenoic acid%	N/A		N/A		N/A	N/A	N/A	N/A
∑ Saturated fatty acids	18.64		21.63		15.51	15.93	23.98	23.10
∑ Monounsaturated fatty acids	10.71		6.86		12.33	10.05	23.33	23.21
∑ Polyunsaturated fatty acids	70.66		73.8		72.05	74.04	52.92	53.76
omega-6: omega-3	16		10		7	7	24	19
Data are the mean ± standard deviation of four replicates. Different letters in the same row (^a^ and ^b^; whole grains and sprouts) indicate significant differences (p < 0.05). N/A means not applicable since fatty acids are not present and therefore cannot be quantitated.								

## Data Availability

The data used to support the findings of this study can be made available by the corresponding author upon request.
